# Fossil skulls reveal that blood flow rate to the brain increased faster than brain volume during human evolution

**DOI:** 10.1098/rsos.160305

**Published:** 2016-08-31

**Authors:** Roger S. Seymour, Vanya Bosiocic, Edward P. Snelling

**Affiliations:** 1 School of Biological Sciences, University of Adelaide, Adelaide, South Australia 5005, Australia; 2 Brain Function Research Group, School of Physiology, University of the Witwatersrand, Johannesburg, Gauteng 2193, South Africa

**Keywords:** brain perfusion, hominin, evolution, cognition, cerebral cortex

## Abstract

The evolution of human cognition has been inferred from anthropological discoveries and estimates of brain size from fossil skulls. A more direct measure of cognition would be cerebral metabolic rate, which is proportional to cerebral blood flow rate (perfusion). The hominin cerebrum is supplied almost exclusively by the internal carotid arteries. The sizes of the foramina that transmitted these vessels in life can be measured in hominin fossil skulls and used to calculate cerebral perfusion rate. Perfusion in 11 species of hominin ancestors, from *Australopithecus* to archaic *Homo sapiens*, increases disproportionately when scaled against brain volume (the allometric exponent is 1.41). The high exponent indicates an increase in the metabolic intensity of cerebral tissue in later *Homo* species, rather than remaining constant (1.0) as expected by a linear increase in neuron number, or decreasing according to Kleiber's Law (0.75). During 3 Myr of hominin evolution, cerebral tissue perfusion increased 1.7-fold, which, when multiplied by a 3.5-fold increase in brain size, indicates a 6.0-fold increase in total cerebral blood flow rate. This is probably associated with increased interneuron connectivity, synaptic activity and cognitive function, which all ultimately depend on cerebral metabolic rate.

## 1. Introduction

The most distinctive feature of modern *Homo sapiens* is the relatively large size of the brain and its high metabolic rate. Across evolutionary history, the hominin brain has undergone increases in size [1,2] and reorganization associated with cognitive specialization [3,4]. To explain the drivers for hominin brain evolution, an emphasis has been placed on understanding hominin cerebral metabolic evolution. The human body allocates 20–25% of total resting metabolic rate to brain function, compared with 8–10% for non-human primates and 3–5% for most non-primate mammals [5–7]. Hominin cerebral metabolic evolution has been proposed to relate to changes in neuronal function and the establishment of specialized communication and metabolic energy pathways [8].

The brain is an entirely aerobic organ that does not store glucose or much glycogen, and so relies on a constant blood supply. Glucose is the prime metabolic fuel and a substrate for biosynthesis [9,10], while oxygen is necessary for oxidative phosphorylation that produces ATP for neuronal and synaptic functions. Although energy is used for diverse cellular activities in the brain, and metabolic rates can shift dramatically between regions of the cerebrum in the short term [11], the overall blood flow rate (perfusion) and metabolic rate of the cerebrum changes little between rest, high cognitive activity, physical exercise and sleep [12–14]. Furthermore, *in vivo* rates of cerebral blood flow, oxygen consumption and glucose uptake scale almost identically with brain volume among mammals, with interspecific exponents of the allometric power equation ranging between 0.82 and 0.87 [9,15–17]. The exponents are lower than direct proportionality (1.0), but greater than the three-quarter exponent (0.75) predicted by the empirical relationship between resting metabolic rate and whole body mass of mammals, known as ‘Kleiber's Law’ [18]. In the primate order, however, neuron numbers increase linearly with brain mass while the volume of individual neurons remains constant [19]. These findings imply that the human brain could be, at a neurological and metabolic level, simply a linearly scaled-up version of the primate brain [20]. Indeed, our estimate of cerebral blood flow rate in 34 species of haplorrhine primates, scales with brain volume to the 0.95 power, which is not significantly different from 1.0 [21]. However, the scaling of brain perfusion in living mammals or primates might not represent the evolution of brain perfusion in hominins.

We use the lumen radius of the internal carotid arteries (ICAs) to deduce changes in cerebral brain metabolism throughout hominin evolution, with the understanding that metabolic rate, blood flow rate and arterial size are tightly related. In haplorrhine primates, including hominins, the perfusion of blood to the cerebrum, the specialized region of the brain responsible for cognitive function, is almost exclusively derived from two ICAs that pass through the carotid canals in the petrous parts of the temporal bones [22,23]. In humans, the ICAs give rise to the middle cerebral arteries that service the lateral parts of the frontal, parietal and temporal lobes, and the anterior cerebral arteries that service the medial parts of the frontal and parietal lobes. The paired vertebral arteries join to form the basilar artery that services the occipital lobes, cerebellum and brain stem. According to data for the radii of these arteries [24], the ICAs supply 85% of total brain blood flow and the basilar artery supplies 15%. These arteries potentially communicate via the Circle of Willis. However, flow through the posterior communicating arteries of the Circle in normal humans can occur in either direction and with velocities that are similar in individuals with bilateral or unilateral vessels [25], and the rate of flow is certainly less than 10% of total brain perfusion, based on their radii [24]. This information indicates that the ICAs are nearly the exclusive blood supply to the cerebrum, and the vertebral and basilar arteries normally play almost no role. Rarely, cerebral perfusion can occur through several collateral arteries if the ICAs are congenitally reduced or absent, and in such cases, the carotid canals in the skull are also reduced or absent [26,27].

The lumen size and wall thickness of large arteries are dynamically controlled throughout life by blood flow requirements and blood pressure [28]. The relationships are so well known as to be ‘laws’ [29]. Wall thickness approximately conforms to the Law of Laplace, in which thickness is proportional to radius and internal pressure [30], and arterial size conforms to Murray's Law that reduces the energy required for circulation [31]. For example, if blood flow rate in the rat common carotid artery is reduced by 35% experimentally, the inner radius of the artery decreases over several weeks to within 11% of the theoretical value derived from the shear stress equation (see Material and methods) that normalizes the frictional force of flowing blood acting on the endothelial lining of the arterial wall [32]. Because the ICAs are not accompanied by significantly sized veins or nerves [33], and they pass snugly through the carotid canal [34], it is possible to estimate blood flow rate from the radius of the carotid foramen in the skull. Using this technique, we estimate total cerebral blood flow rate via the left and right ICAs (${\dot{Q}}_{\mathrm{ICA}}$; cm^3 ^s^−1^), from the size of the internal carotid foramina of 35 fossil specimens from 12 hominin species. We use this as a proxy for cerebral metabolic rate and cognitive evolution, assuming that the fraction of oxygen extracted from the blood is size-independent, as it is in the whole body of resting mammals [35]. We then scale ${\dot{Q}}_{\mathrm{ICA}}$ against body size, brain volume and fossil age to show the evolutionary progression of the hominin brain.

## 2. Material and methods

### 2.1. Carotid foramina dimensions

**Figure 1.**
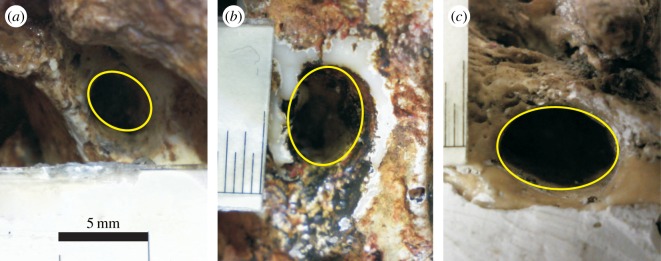
Internal carotid foramina in selected hominin species: (*a*) *Australopithecus africanus*, (*b*) *Homo neanderthalensis* (cast) and (*c*) archaic *Homo sapiens*. All photographs are the same scale (increments in *b* and *c* are 0.5 mm) and thus illustrate the increase in foramen size across hominin evolution.

We measured the dimensions of the carotid foramen, the external opening of the carotid canal, which allows passage of the ICA into the cavity of the skull (figure 1). Carotid canal dimensions were measured from the skulls of 35 specimens of 12 species of hominin including *Australopithecus africanus* (*N* = 8 individuals), *A. afarensis* (*N* = 3), *A. boisei* (*N* = 1), *Homo habilis* (*N* = 1), *H. naledi* (*N* = 1), *H. rudolfensis* (*N* = 1), *H. georgicus* (*N* = 1), Early and Late *H. erectus* (*N* = 5), *H. heidelbergensis* (*N* = 2), *H. neanderthalensis* (*N* = 5), *H. floresiensis* (*N* = 1) and archaic *H. sapiens* (*N* = 5). Specimens were obtained from the South Australian Museum (Adelaide, Australia), the J. L. Shellshear Museum of Physical Anthropology and Comparative Anatomy (University of Sydney, Sydney, Australia), the Evolutionary Studies Institute (University of the Witwatersrand, Johannesburg, South Africa) and the Ditsong National Museum of Natural History (Pretoria, South Africa). See the electronic supplementary material for the specimen inventory.

Original skulls were selected based on the preserved integrity of the foramen of the carotid canal. Only complete and undamaged foramina were included in the study. The edges of the foramina were intact and the interior surfaces were smooth, free of sediment or crystals, and showed no significant signs of degradation. Most skulls and casts presented only one morphologically intact foramen. If two were present, the best preserved foramen was analysed. Skull casts were used when original skulls were unavailable and were also selected based on the integrity of the carotid foramen. In some instances, it was possible to compare casts with original fossils of the same specimen. The foramen of the cast compared with the original matched within 50 µm.

The carotid foramina were photographed as close to perpendicular to the opening as possible. A digital microscope (ViTiny Pro10 Plus) allowed measurement of the diameter of the opening to a precision of 50 µm. A scale bar was placed on the temporal bone adjacent to the foramen and as close as possible to the level of the foramen to eliminate parallax effect (figure 1). Internal carotid foramen area was measured with respect to the scale using ImageJ (www.nih.gov). As the carotid foramina were slightly elliptical, the minimum and maximum diameters were used to calculate the area of the ellipse, and the radius of a circle of that area was taken as foramen radius. The radius of the arterial lumen then was calculated, given that vessel wall thickness is proportional to lumen radius according to the Law of Laplace. The ratio of wall thickness to lumen radius (0.4 : 1) was derived from the carotid arteries of three mammalian species, including humans [21]. Blood flow rate ($\dot{Q}$; cm^3 ^s^−1^) was calculated according to the equation, $\dot{Q}=(\tau \pi r^{3})/4\eta$, where *τ* is shear stress (dyne cm^−2^), *r* is arterial lumen radius (cm) and *η* is blood viscosity (dyne s cm^−2^) [36]. This equation is independent of blood pressure. Blood viscosity is assumed to be constant, 0.04 dyne s cm^−2^, as calculated for mammalian large blood vessels [37]. Shear stress in the ICA was calculated according to the equation, $\tau =167M_{b}^{-0.20}$, which we derived from empirical data in humans and rats, and where *M*_b_ is body mass in grams [21]. The exponent of this equation is virtually identical to −0.21 derived for the common carotid arteries of mice, rats, rabbits and humans [38]. A full validation of these calculations can be found in reference [21]. Briefly, ICA lumen radius calculated from carotid foramen radius in seven recent human skulls, and ICA lumen radius calculated from direct measures of ICA blood flow rate in humans (four studies using phase contrast quantitative magnetic resonance angiography, positron emission tomography and electromagnetic flow meters) agreed within 10% of each other. Rodents are another mammalian group in which the ICAs provide the major cerebral blood supply. Once again, the agreement between ICA lumen radius calculated from published blood flow rates and that calculated from actual foramen radius, is within 10% for mice and less than 1% for rats.

### 2.2. Statistical analyses

Total ICA blood flow rate, ${\dot{Q}}_{\mathrm{ICA}}$ (cm^3 ^s^−1^), was calculated by doubling the value derived for one carotid foramen for each hominin specimen or cast. An average ${\dot{Q}}_{\mathrm{ICA}}$ was then obtained for each species. Estimates of adult body mass (*M*_b_) and endocranial brain volume (*V*_br_) were obtained from the literature for each individual specimen; when unavailable, mean values for the species were used. All data were analysed using the power equation, *Y* = *aX^*b*^*, where *Y* is the variable of interest, *X* is body mass or endocranial volume, *a* is the scaling factor (elevation of the curve) and *b* is the scaling exponent (shape of the curve). *Y* is proportionate to *X* only if *b* = 1.0; if 1 > *b *> 0, the arithmetic curve increases with a decreasing slope and the ratio of *Y*/*X* decreases; if *b* > 1, the arithmetic curve increases with an increasing slope and the ratio of *Y*/*X* increases. The power equation was derived from ordinary least squares regressions of log-transformed data of species means, and 95% confidence intervals for the exponents were calculated with StatistiXL v. 1.8 (www.statistixl.com). 95% confidence bands for the regression means were calculated with GraphPad Prism 6 (www.graphpad.com). ${\dot{Q}}_{\mathrm{ICA}}$ was related to the published geological age (Ma) of each species with a second-order polynomial regression in Microsoft Excel.

## 3. Results

**Figure 2.**
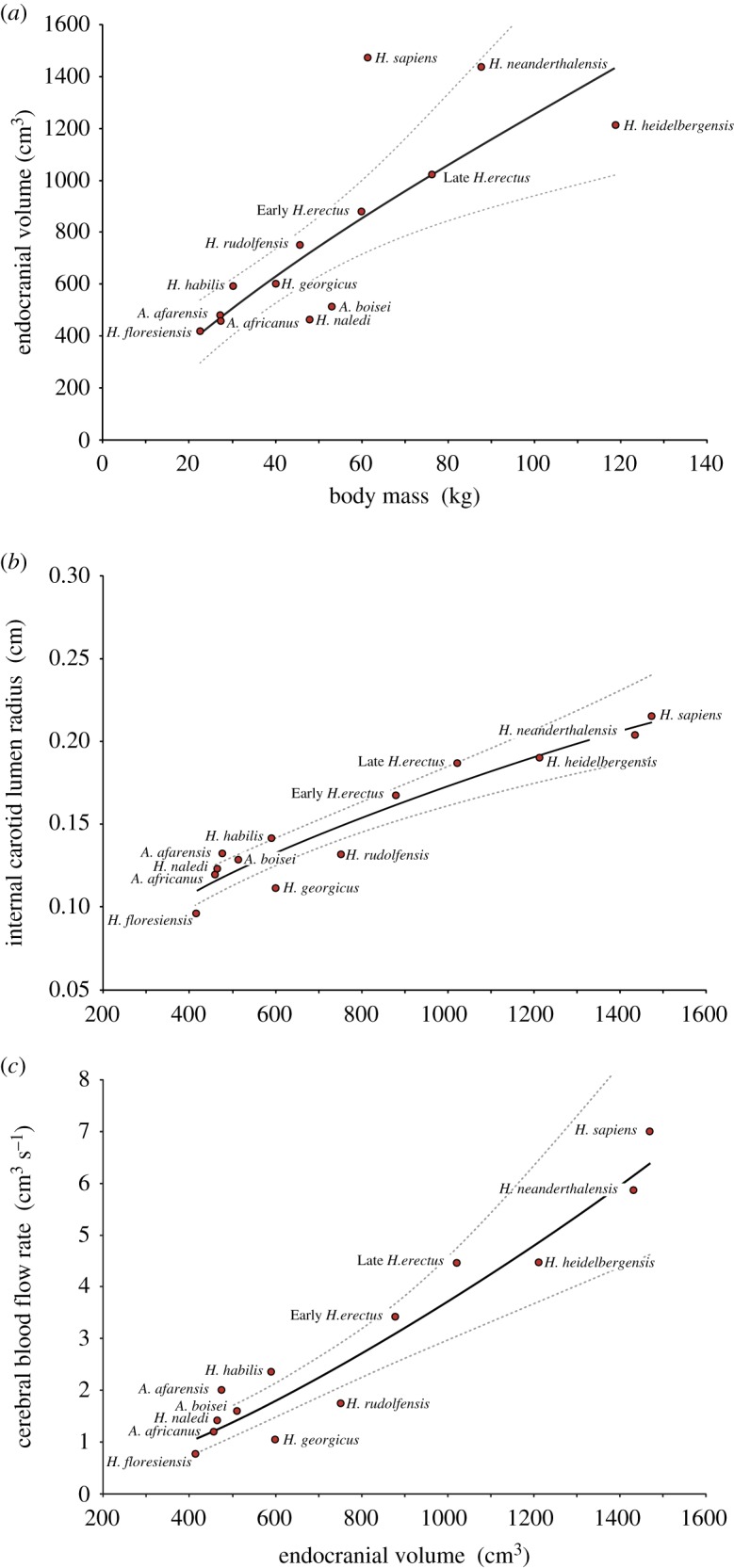
Allometric relationships derived for the brains of 12 species of hominins: (*a*) mean endocranial volume (*V*_br_) plotted against body mass (*M*_b_), where $V_{\mathrm{br}}=37.9M_{b}{\;}^{0.76}$. (*b*) Mean lumen radius (*r*) of internal carotid arteries in relation to endocranial volume (*V*_br_), where $r=4.79\times 10^{-3}\;V_{\mathrm{br}}{\;}^{0.52}$. (*c*) Cerebral blood flow rate (${\dot{Q}}_{\mathrm{ICA}}$), calculated from the size of the internal carotid foramina, in relation to endocranial volume (*V*_br_), where $\dot{Q}{\;}_{\mathrm{ICA}}=2.10\times 10^{-4}\;V_{\mathrm{br}}{\;}^{1.41}$. Allometric power regressions (solid curves) with 95% confidence bands (dashed curves) are presented on arithmetic axes, but were calculated on log-transformed data.

Hominin endocranial volume (*V*_br_, cm^3^) increases with body mass (*M*_b_, kg) according to the allometric power equation $V_{\mathrm{br}}=37.9M_{b}{\;}^{0.76}$ (*R*^2^ = 0.69; *p* < 0.001) (figure 2*a*). The 95% confidence interval of the exponent is ±0.33, indicating a value not significantly different from 1.0, which is the exponent for isovolumetric expansion. The broad confidence interval is partly related to *H. sapiens*, which is a clear and statistically significant outlier, possessing a much larger brain relative to body mass compared with other hominin species. Nevertheless, the exponent of 0.76 is similar to that derived for haplorrhine primates (0.71) and diprotodont marsupials (0.70) [21].

Internal carotid lumen radius (*r*, cm) increases with endocranial volume according to the equation $r=4.79\times 10^{-3}\;V_{\mathrm{br}}{\;}^{0.52}$ (*R*^2^ = 0.87; *p* < 0.001) (figure 2*b*). The 95% confidence interval of the exponent is ±0.13, indicating a value significantly greater than 0.33, which is the exponent expected for a linear dimension of a volume increasing in size with the same shape. Because lumen radius is proportional to internal carotid foramen radius, the scaling of foramen radius alone indicates that brain perfusion increases disproportionately with brain volume.

Total ICA blood flow rate (${\dot{Q}}_{\mathrm{ICA}}$; cm^3 ^s^−1^) increases with endocranial volume according to the equation $\dot{Q}{\;}_{\mathrm{ICA}}=2.10\times 10^{-4}\;{V_{\mathrm{br}}}^{1.41}$ (*R*^2^ = 0.85; *p* < 0.001) (figure 2*c*). The 95% confidence interval of the exponent is ±0.39, indicating a value significantly greater than 1.0, which is the exponent expected if brain perfusion were linearly related to brain size. Instead, ${\dot{Q}}_{\mathrm{ICA}}$ increases 6.0-fold (from 1.07 to 6.37 cm^3 ^s^−1^) as *V*_br_ increases 3.5-fold over its entire range (from 417 to 1471 cm^3^). Thus, the volume-specific cerebral perfusion rate increases 1.7-fold, from 2.57 to 4.33 cm^3 ^s^−1 ^l^−1^.

**Figure 3.**
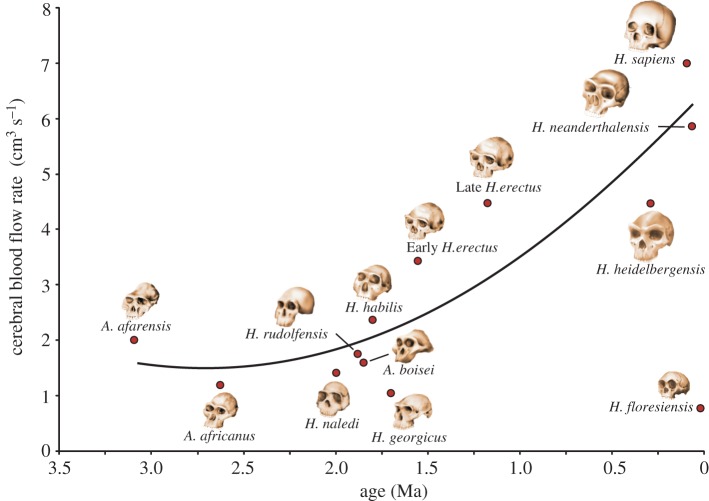
Cerebral blood flow rate (${\dot{Q}}_{\mathrm{ICA}}$) in relation to estimated geological age (*A*) in 12 hominin species, where ${\dot{Q}}_{\mathrm{ICA}}=0.677A^{2}-3.69A+6.52$. *Homo floresiensis* is excluded from the regression.

The increase in ${\dot{Q}}_{\mathrm{ICA}}$ occurred during the progression of geological age (*A*; Ma) and is described by the second-order polynomial equation ${\dot{Q}}_{\mathrm{ICA}}=0.677A^{2}-3.69A+6.52\;(R^{2}=0.84)$ (figure 3). The low datum from *H. floresiensis* is omitted from the regression as it is an obvious outlier.

## 4. Discussion

Our research shows that total ICA blood flow rate, which appears to be proportional to cerebral metabolic rate, scales with a disproportionately steep exponent of 1.41 relative to endocranial volume (figure 2*c*). This exponent is significantly greater than 0.75 and, therefore, does not support the hypothesis that hominin brain metabolism conforms to ‘Kleiber's Law’ for organs [18]. Furthermore, the exponent is significantly greater than 1.0 and so does not support the hypothesis of a proportional increase in brain metabolism relative to endocranial volume. We suggest that the disproportionate increase in cerebral blood flow rate during hominin evolution reflects an increasing metabolic intensity of cerebral tissue associated with evolutionary reorganization of the brain. Several lines of evidence, including slow postnatal neural development and upregulation of genes involved in glucose metabolism [39] suggest that humans have elevated brain metabolic rate compared with other primates.

Communication pathways among neurons in the hominin brain are assumed to have undergone significant changes throughout evolution. The relative volume of cerebral prefrontal white matter is significantly larger in humans than in non-human primates [40]. The high proportion of white matter in the human brain, and the high exponent of cerebral perfusion on brain volume, suggest that increasing interneuron connectivity augmented the metabolic demands of the evolving brain. The steep increase of cerebral perfusion may be associated with increased neuronal communication and establishment of cerebral left–right hemisphere specialization [41], both of which are associated with selection for cognitive advancement [42].

Synaptic transmission accounts for more than one-half of the brain's total metabolic energy requirements [43]. It can, therefore, be inferred that the disproportionate increase in blood flow to the cerebrum throughout hominin evolution is associated with an increase in the number of synapses per neuron or greater synaptic activity. This would have facilitated enhanced information processing and communication pathways as cortical enlargement and regional specialization took place. In support, glutamate, one of the most prevalent neurotransmitters in the cerebral cortex [44], experiences higher turnover rates in the brains of humans compared with chimpanzees [45], which possibly reflects an evolutionary selection for increasingly energy-expensive hominin brains. The complexity and diversity of cortical astrocytes in the adult human brain are unique within the primate order [46]. Astrocytes play a crucial role in neuronal and synaptic energetics [47]. Astrocyte concentrations around neuronal terminals are activity dependent [48], and astrocytes regulate cerebral blood flow by releasing vasoactive substances in response to neuronal activity, resulting in the coupling of regional cerebral perfusion with regional energy demand [49,50]. Our results may be related, in part, to the evolutionary trajectory of astrocyte concentrations within the hominin brain.

Environmental and life-history changes associated with the evolution of late *Homo* would have probably selected for enhanced postnatal neuroplasticity to deal with environmental and biological change, which in turn would have contributed to increased brain asymmetry and regional specialization [51]. Synaptic density in the prefrontal cortex peaks within 4–5 years of age in humans, in comparison with less than 1 year of age in chimpanzees and macaque monkeys [52]. This suggests that extended juvenile development is associated with a greater density of synapses at an age when neuroplasticity is highest. This results in a significantly heightened ability to process and translate information crucial to cognitive development and controls the extent of synaptic loss via pruning.

Increased cerebral metabolism in *Homo* probably co-evolved with increased length of juvenile development. Cerebral metabolic investment in *Homo* infants was probably a strong selection pressure at the expense of early physical maturation, becoming increasingly pronounced with the evolution of *H. sapiens*. Our research further suggests that hominin cerebral blood flow and metabolism may be good indicators of the length of juvenile development and cessation of synaptic development. This is supported by the fact that, although Neanderthals and humans have similar brain volumes (figure 2*a*), Neanderthals exhibit lower cerebral blood flow rates (figure 2*c*). Neanderthals also experienced earlier molar eruptions, indicating earlier maturation [53]. Extended juvenile development would have allowed *Homo* infants to invest more energy into learning, practising and perfecting cognitively complex tasks [54]. This may have selected for more specialized, region-specific, synaptic pruning in the later *Homo* species in comparison with earlier *Homo* and *Australopithecus*.

We believe that analysis of carotid foramen size and endocranial brain volume of fossil skulls provides the only possible estimate of cerebral perfusion rate, metabolic rate and cognitive ability across hominin evolution. Because it is based on actual hominin fossils, it is more direct and reliable than inferences from measurements of brain structure and metabolism in living mammals, which do not represent hominin evolution. Of course, our analysis relies on the presently untestable assumption that the pattern of cerebral perfusion is the same in humans as it was in ancestral hominins. However, the blood supply to the cerebrum of haplorrhine primates is derived mainly from the ICAs [22], and stapedial arteries that branch from the ICAs in the carotid canal are absent in anthropoid apes [55], including humans [56]. This provides confidence that the pattern of cerebral blood flow was established prior to the divergence of *Australopithecus* from the common ancestor among the anthropoid apes and that carotid foramen size is a reliable indication of a pattern of increased cerebral perfusion during hominin evolution.

## Supplementary Material

Data for individual hominin specimens and sources of information

## Data accessibility

A full dataset and specimen inventory is presented in the electronic supplementary material.
