# Correction to ‘Fossil skulls reveal that blood flow rate to the brain increased faster than brain volume during human evolution’

**DOI:** 10.1098/rsos.170846

**Published:** 2017-08-30

**Authors:** Roger S. Seymour, Vanya Bosiocic, Edward P. Snelling

*R. Soc. open sci.* **3**, 160305. (Published 31 August 2016). (doi:10.1098/rsos.160305)

We analysed the size of the internal carotid foramina in relation to endocranial volume in 12 species of hominins and concluded that blood flow rate to the brain increased sixfold, while brain volume increased only 3.5-fold over the last 3 Myr of hominin evolution [1]. The data were based on 34 casts or original fossils. It has come to our attention that three of the casts may not represent the original fossils accurately. These casts were manufactured by Bone Clones (Bone Clones Inc., Canoga Park, CA, USA) and were identified as *Australopithecus afarensis* (BH-021-A), *Australopithecus boisei* (BH-015) and *Homo neanderthalensis* (BH-009). We were unaware that some details of these casts are not based on actual fossils, but are artistic recreations intended for education. Although the casts are nominally attributed to specific fossils, certain details are based on other skulls and the literature. The Bone Clones website indicates clearly that, ‘These are not recommended for advanced research purposes’. In particular, the original skull of *A. afarensis* (A.L. 288-1) on which BH-021-A is based, lacks the entire base of the skull and the carotid foramina [2]. On this basis, we have reanalysed those data without the three Bone Clones' casts. In addition, one cast (Skhul5) in our original analysis was attributed incorrectly to *H. neanderthalensis* instead of *H. sapiens*. The revised dataset is presented in the electronic supplementary material (table S1) accompanying this correction paper.

The revised data involve endocranial brain volume (*V*_br_, cm^3^), mean internal carotid artery (ICA) foramen radius (*r*_F‐ICA_, cm), ICA lumen radius (*r*_L‐ICA_, cm), combined blood flow rate of the two ICAs supplying the brain (${\dot{Q}}_{\mathrm{ICA}},$ cm^3^ s^−1^), and the brain-volume-specific blood flow rate (${\dot{Q}}_{\mathrm{ICA}}/V_{\mathrm{br}},$ cm^3^ s^−1^ l^−1^). They are based on 11 species of hominins, but the sample size for the regressions is 12, because Early *Homo erectus* and Late *H. erectus* are considered separately. The revised allometric power equations are based on ordinary least-squares linear regressions of log-transformed data and are presented with 95% confidence intervals for the exponents as follows:

$$\begin{array}{rl} r_{F\text{-}\mathrm{ICA}} & \;=5.42\times 10^{-3}\;V_{\mathrm{br}}^{0.55\pm 0.14}\;(R^{2}=0.88),\\ r_{L\text{-}\mathrm{ICA}} & \;=3.87\times 10^{-3}\;V_{\mathrm{br}}^{0.55\pm 0.14}\;(R^{2}=0.88),\\ {\dot{Q}}_{\mathrm{ICA}} & \;=1.70\times 10^{-4}\;V_{\mathrm{br}}^{1.45\pm 0.42}\;(R^{2}=0.85)\\ \text{and}\;\;\frac{{\dot{Q}}_{\mathrm{ICA}}}{V_{\mathrm{br}}} & \;=0.170\;V_{\mathrm{br}}^{0.45\pm 0.42}\;(R^{2}=0.36). \end{array}$$

The allometric equations set to the data here are slightly changed from the original paper [1]. The exponents for radii increase from 0.52 to 0.55, and that for ${\dot{Q}}_{\mathrm{ICA}}$ increases slightly from 1.41 to 1.45 (figure 1). The loss of *A. boisei* is unnoticed, because its former data point was very close to the regression line. Compared with the remaining data for ICA foramen radius measured from the same species and the same way [1], the Bone Clone BH-021-A is 7% higher than the mean for other *A. afarensis* specimens, and BH-009 is only 2% lower than the mean for other *H. neanderthalensis* specimens. If only original fossil specimens (no casts at all) are included in the analysis, the exponents are still very high (*r*_F‐ICA_ = 8.18 × 10^−3^ *V*_br_^0.50^ and ${\dot{Q}}_{\mathrm{ICA}}=4.02\times 10^{-4}\;{V_{\mathrm{br}}}^{1.35}$). Data from the original fossils include *A. africanus*, *A. afarensis* and archaic *H. sapiens*, and these species offer the most leverage for the regressions.

**Figure 1.**
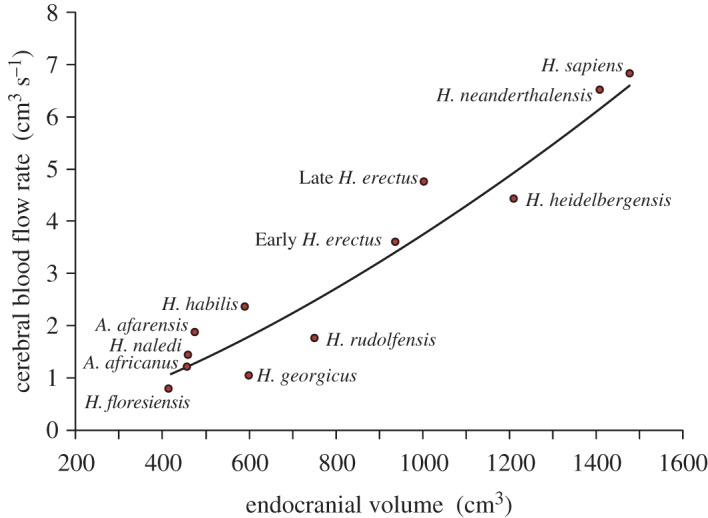
Cerebral blood flow rate (${\dot{Q}}_{\mathrm{ICA}}$) in relation to endocranial volume (*V*_br_) for 11 species of hominins. The curve represents the allometric equation given in the text.

The original and revised exponents are very much higher than expected from isometry based on brain size (i.e. $\mathrm{radius}\propto V_{\mathrm{br}}^{0.33}$ and ${\dot{Q}}_{\mathrm{ICA}}\propto V_{\mathrm{br}}^{1.0}$), showing that brain perfusion increased disproportionately with brain volume and hence, the metabolic intensity of brain tissue increased during the course of hominin evolution. The original paper considered only total blood flow rates to the enlarging brain. Here, we take the opportunity to present brain-volume-specific blood flow rates to provide estimates of the metabolic intensity of the tissue. Over all, the allometric equation shows that the intensity increases 1.7-fold over the range of brain volume (taken as 458 ml in *A. africanus* to 1471 ml in *H. sapiens*; figure 2). This is the same conclusion as in our original paper, which suggests that the increase was due to greater synaptic energy consumption in later *Homo* species. This may have been associated with increased proportion of grey matter that has a metabolic intensity about twice that of white matter [3]. However, there is considerable scatter in the volume-specific data (figure 2), and it is dangerous to form conclusions about individual species until much greater sample sizes are available.

**Figure 2.**
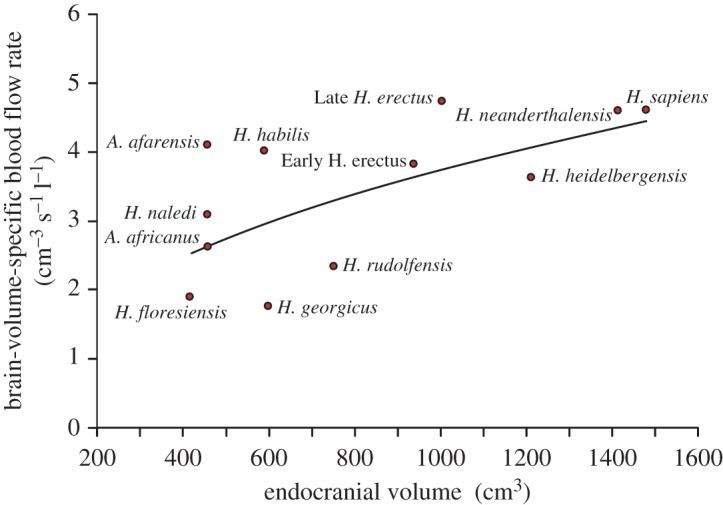
Brain-volume-specific blood flow rate (${\dot{Q}}_{\mathrm{ICA}}/V_{\mathrm{br}}$) in relation to endocranial volume (*V*_br_) for 11 species of hominins. The curve represents the allometric equation given in the text.

The original paper provides estimates of the ages of the hominin species from the literature to show the evolution of brain blood flow rates. There may be disagreement in the literature about fossil ages, but the errors are not large, with one exception, *Homo naledi*. The initial estimate of its age was about 2 Ma, but new data estimate a much younger age, 0.285 Ma [4]. If the rate of blood flow is considered to represent the metabolic rate of the cerebral tissue, and metabolic rate is related to cognitive ability, then it appears that both *Homo floresiensis* and *H. naledi* had brain functions more similar to *Australopithecus* than to more recent *Homo* species. The revised relationship between ${\dot{Q}}_{\mathrm{ICA}}$ and nominal age shows a general trend for exponentially increasing perfusion among the other species (figure 3); however, this does not imply direct descendancy.

**Figure 3.**
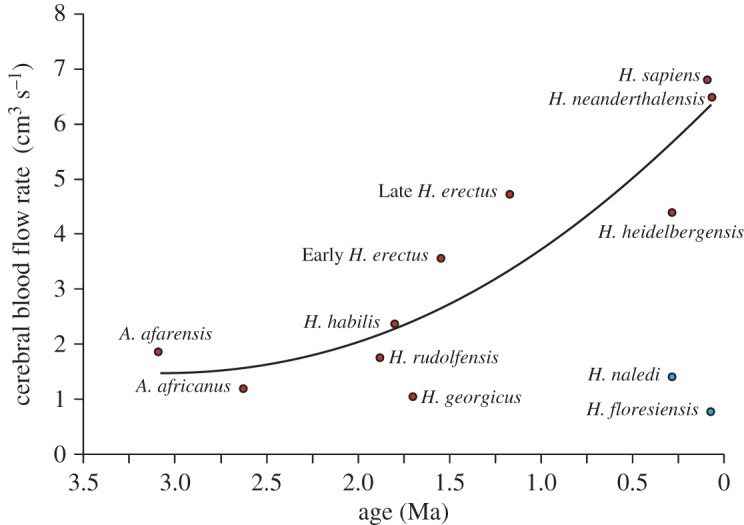
Cerebral blood flow rate (${\dot{Q}}_{\mathrm{ICA}}$) in relation to estimated geological age (*A*) in nine hominin species, where ${\dot{Q}}_{\mathrm{ICA}}=0.576A^{2}-3.436A+6.61\;(R^{2}=0.82)$. *Homo floresiensis* and *H. naledi* are excluded from the regression.

## Supplementary Material

Data for individual hominin fossil skulls

## Data accessibility

All data are presented in an electronic supplementary material, table S1, associated in the journal with this paper.
