# Supplementary material for: Fossil skulls reveal that blood flow rate to the brain increased faster than brain volume during human evolution
Source: R Soc Open Sci. 2016 Aug 31;3(8):160305. doi: 10.1098/rsos.160305 (PMC5108958; doi:10.1098/rsos.160305)
Supplement: Data for individual hominin specimens and sources of information [file rsos160305supp1.docx]

**Fossil skulls reveal that blood flow rate to the brain increased faster than brain volume during human evolution**

**Roger S. Seymour^,^, Vanya Bosiocic and Edward P. Snelling**

**Supplementary Material**

**Sources of data for individual specimens**


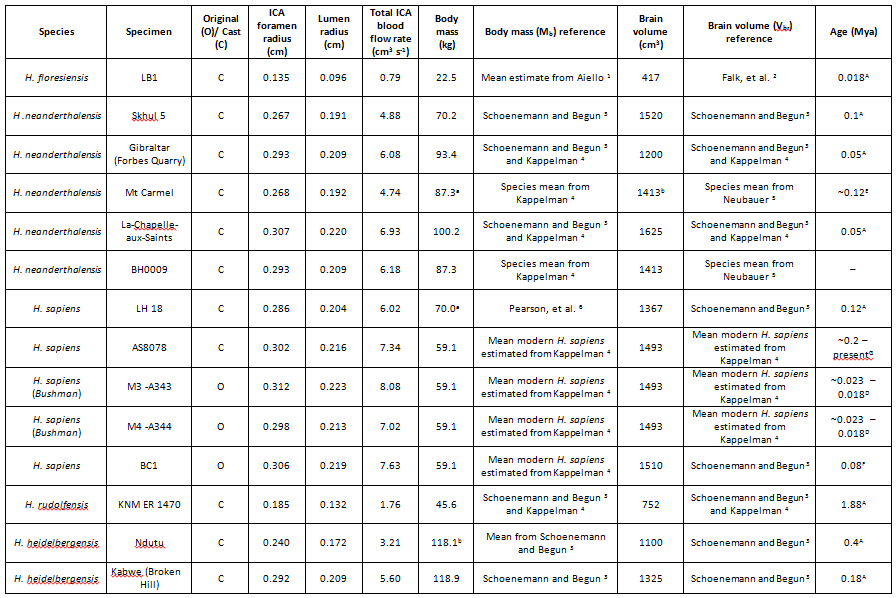


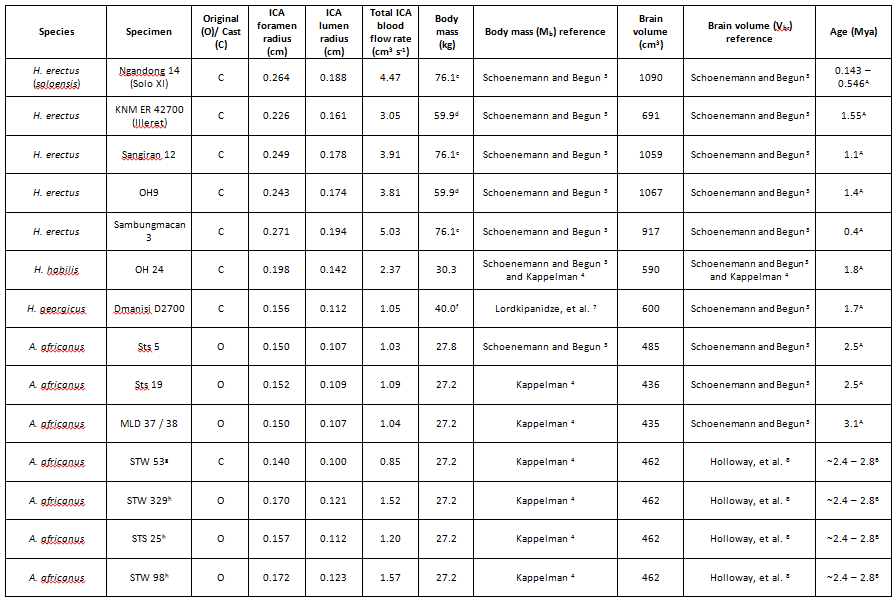


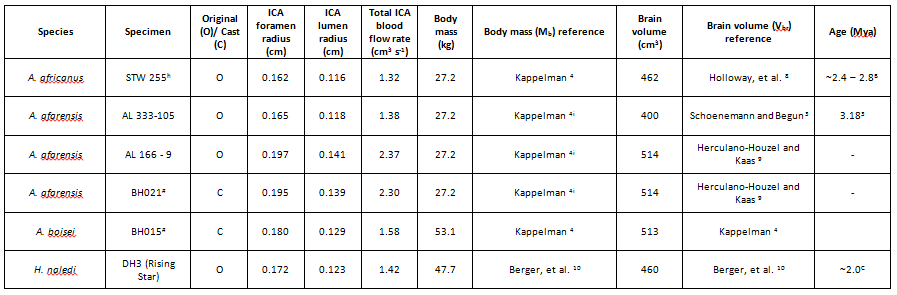


^a^ body mass of LH 18 derived from Omo 1 due to its physiological resemblance as noted by Magori and Day ^11^.

^b^ Ndutu individual was based in Tanzania, and so allocated body mass was estimated by averaging the known body masses of two African *H. heidelbergensis* individuals (Kebwe and Bodo).

^c^ body mass of *H. erectus soloensis* estimated from Sangiran 17 (due to lack of published data), which shares a common endocrinal volume, geographical habitation and age.

^d^ body mass estimated from known body mass of KNM – WT 15000, which shared common age and geographical habitation, although it is noted that KNM ER 42700 (IIIeret) does have significantly smaller endocrinal volume in comparison to mean endocrinal volume for *H. erectus*.

^f^ lower estimated endocrinal volume used owing to subadult developmental stage of Dmanisi D2700 as recommended by [Lordkipanidze *et al.* (2007](#_ENREF_5)).

^g^ speciation determined from Kuman and Clarke ^12^ and Clarke ^13^.

^h^ temporal bone specimen speciated from Braga, et al. ^14^.

^I^ body mass of *A. afarensis* derived from body mass estimate of *A. africanus*, as Jungers ^15^ states mininal differences in body size between *Australopithecus* taxa.

^A^ specimen age determined from [Schoenemann and Begun ^3^](#_ENREF_3) .

^B^ specimen collection from Sterkfontein Member 4 ^16^, and age of specimens identified from McKee, et al. ^17^.

^C^ specimen age approximated from Berger *et al* ^10^.

^D “^Bushman – oids” specimens of Mumbwa strata IV aged according to Protsch ^18^.

^E^ specimen age according to [Mercier *et al.* (1993](#_ENREF_12)).

^F^ specimen age according to Lahr ^19^.

^G^ mean species age according to Trinkaus ^20^.

^#^ cast number from the South Australian Museum.

**Supplementary References**

1 Aiello, L. C. Five years of *Homo floresiensis*. *Am. J. Phys. Anthropol.* **142**, 167-179 (2010).

2 Falk, D. *et al.* Brain shape in human microcephalics and *Homo floresiensis*. *Proc. Natl. Acad. Sci. U. S. A.* **104**, 2513-2518 (2007).

3 Schoenemann, P. T. & Begun, D. in *A Companion to Paleoanthropology* 136-164 (Blackwell Publishing Ltd., 2013).

4 Kappelman, J. The evolution of body mass and relative brain size in fossil hominids. *J. Hum. Evol.* **30**, 243-276 (1996).

5 Neubauer, F. A brief overview of the last 10 years of major late pleistocene discoveries in the Old World: *Homo floresiensis*, neanderthal, and denisovan. *Journal of Anthropology* **2014**, 7 (2014).

6 Pearson, O. M., Royer, D. F., Grine, F. E. & Fleagle, J. G. A description of the Omo I postcranial skeleton, including newly discovered fossils. *J. Hum. Evol.* **55**, 421-437 (2008).

7 Lordkipanidze, D. *et al.* Postcranial evidence from early *Homo* from Dmanisi, Georgia. *Nature* **449**, 305-310 (2007).

8 Holloway, R. L., Broadfield, D. C. & Yuan, M. S. in *The Human Fossil Record* 295-301 (John Wiley & Sons, Inc., 2005).

9 Herculano-Houzel, S. & Kaas, J. H. Gorilla and orangutan brains conform to the primate cellular scaling rules: implications for human evolution. *Brain, behavior and evolution* **77**, 33-44 (2011).

10 Berger, L. R. *et al.* *Homo naledi*, a new species of the genus *Homo* from the Dinaledi Chamber, South Africa. *Elife* **4**, e09560 (2015).

11 Magori, C. C. & Day, M. H. Laetoli Hominid 18: an early *Homo sapiens* skull. *J. Hum. Evol.* **12**, 747-753, doi:http://dx.doi.org/10.1016/S0047-2484(83)80130-4 (1983).

12 Kuman, K. & Clarke, R. J. Stratigraphy, artefact industries and hominid associations for Sterkfontein, Member 5. *J. Hum. Evol.* **38**, 827-847 (2000).

13 Clarke, R. Latest information on Sterkfontein's *Australopithecus* skeleton and a new look at *Australopithecus*. *South African Journal of Science* **104**, 443-449 (2008).

14 Braga, J. *et al.* A new partial temporal bone of a juvenile hominin from the site of Kromdraai B (South Africa). *J. Hum. Evol.* **65**, 447-456 (2013).

15 Jungers, W. L. New estimates of body size in australopithecines. *Evolutionary history of the “robust” australopithecines*, 115-125 (1988).

16 Pickering, T. R., Clarke, R. J. & Moggi-Cecchi, J. Role of carnivores in the accumulation of the Sterkfontein Member 4 hominid assemblage: A taphonomic reassessment of the complete hominid fossil sample (1936–1999). *Am. J. Phys. Anthropol.* **125**, 1-15, doi:10.1002/ajpa.10278 (2004).

17 McKee, J. K., Thackeray, J. F. & Berger, L. R. Faunal assemblage seriation of southern African Pliocene and Pleistocene fossil deposits. *Am. J. Phys. Anthropol.* **96**, 235-250 (1995).

18 Protsch, R. Mumbwa: Its absolute chronology and archaeology. *Z. Morphol. Anthropol.* **68**, 1-7 (1977).

19 Lahr, M. M. *Genetics and fossil evidence from modern human origins*. (Oxford University Press, 2013).

20 Trinkaus, E. Early modern humans. *Annu. Rev. Anthropol.* **34**, 207-230, doi:10.1146/annurev.anthro.34.030905.154913 (2005).
