# Supplementary material for: Correction to ‘Fossil skulls reveal that blood flow rate to the brain increased faster than brain volume during human evolution’
Source: R Soc Open Sci. 2017 Aug 30;4(8):170846. doi: 10.1098/rsos.170846 (PMC5579132; doi:10.1098/rsos.170846)
Supplement: Data for individual hominin fossil skulls [file rsos170846supp1.docx]

**Table S1**. Revised data from Correction to ‘Fossil skulls reveal that blood flow rate to the brain increased faster than brain volume during human evolution’. by Roger S. Seymour, Vanya Bosiocic, & Edward P. Snelling.

| **Species** | **Specimen** | **Original (O) / Cast (C)** | **ICA foramen radius (cm)** | **Lumen radius (cm)** | **Total ICA blood flow rate (cm^3^ s^-1^)** | **Body mass (kg)** | **Body mass (M_b_)**  **reference** | **Brain volume (cm^3^)** | **Brain volume (V_br_) reference** | **Age**  **(Mya)** |
| --- | --- | --- | --- | --- | --- | --- | --- | --- | --- | --- |
| *H. floresiensis* | LB1 | C | 0.135 | 0.096 | 0.79 | 22.5 | Mean estimate from Aiello ^1^ | 417 | Falk, *et al*. ^2^ | 0.06 – 0.10^H^ |
| *H. neanderthalensis* | Gibraltar (Forbes Quarry) | C | 0.293 | 0.209 | 6.08 | 93.4 | Schoenemann ^3^ and Kappelman ^4^ | 1200 | Schoenemann ^3^ and Kappelman ^4^ | 0.05^A^ |
| *H. neanderthalensis* | La Chapelle-aux-Saints | C | 0.307 | 0.220 | 6.93 | 100.2 | Schoenemann ^3^ and Kappelman ^4^ | 1625 | Schoenemann ^3^ and Kappelman ^4^ | 0.05^A^ |
| *H. sapiens* | AS8078 | C | 0.302 | 0.216 | 7.34 | 59.1 | Mean modern *H. sapiens* estimated from Kappelman ^4^ | 1493 | Mean modern *H. sapiens* estimated from Kappelman ^4^ | ~0.2 – present^G^ |
| *H. sapiens* (*Bushman*) | M3-A343 | O | 0.312 | 0.223 | 8.08 | 59.1 | Mean modern *H. sapiens* estimated from Kappelman ^4^ | 1493 | Mean modern *H. sapiens* estimated from Kappelman ^4^ | ~0.023 – 0.018^D^ |
| *H. sapiens* (*Bushman*) | M4-A344 | O | 0.298 | 0.213 | 7.02 | 59.1 | Mean modern *H. sapiens* estimated from Kappelman ^4^ | 1493 | Mean modern *H. sapiens* estimated from Kappelman ^4^ | ~0.023 – 0.018^D^ |
| *H. sapiens* | BC1 | O | 0.306 | 0.219 | 7.63 | 59.1 | Mean modern *H. sapiens* estimated from Kappelman ^4^ | 1510 | Schoenemann ^3^ | 0.08^F^ |
| *H. sapiens* | LH 18 | C | 0.286 | 0.204 | 6.02 | 70.0^a^ | Pearson, *et al*. ^6^ | 1367 | Schoenemann ^3^ | 0.12^A^ |
| *H. sapiens* | Skhul 5 | C | 0.268 | 0.192 | 4.74 | 70.1 | Schoenemann ^3^ | 1520 | Mean modern *H. sapiens* estimated from Kappelman ^4^ | ~0.120 – 0.080^C^ |
| *H. rudolfensis* | KNM-ER 1470 | C | 0.185 | 0.132 | 1.76 | 45.6 | Schoenemann ^3^ and Kappelman ^4^ | 752 | Schoenemann ^3^ and Kappelman ^4^ | 1.88^A^ |
| *H. heidelbergensis* | Ndutu | C | 0.240 | 0.172 | 3.21 | 118.1^b^ | Mean from Schoenemann ^3^ | 1100 | Schoenemann ^3^ | 0.4^A^ |
| *H. heidelbergensis* | Kabwe (Broken Hill) | C | 0.292 | 0.209 | 5.60 | 118.9 | Schoenemann ^3^ | 1325 | Schoenemann ^3^ | 0.18^A^ |
| *H. erectus* (*soloensis*) | Ngandong 14 (Solo XI) | C | 0.264 | 0.188 | 4.47 | 76.1^c^ | Schoenemann ^3^ | 1090 | Schoenemann ^3^ | 0.143 – 0.546^A^ |
| *H. erectus* | KNM-ER 42700 (IIIeret) | C | 0.226 | 0.161 | 3.05 | 59.9^d^ | Schoenemann ^3^ | 691 | Schoenemann ^3^ | 1.55^A^ |
| *H. erectus* | Sangiran 12 | C | 0.249 | 0.178 | 3.91 | 76.1^c^ | Schoenemann ^3^ | 1059 | Schoenemann ^3^ | 1.1^A^ |
| *H. erectus* | OH 9 | C | 0.243 | 0.174 | 3.81 | 59.9^d^ | Schoenemann ^3^ | 1067 | Schoenemann ^3^ | 1.4^A^ |
| *H. erectus* | Sambungmacan 3 | C | 0.271 | 0.194 | 5.03 | 76.1^c^ | Schoenemann ^3^ | 917 | Schoenemann ^3^ | 0.4^A^ |
| *H. habilis* | OH 24 | C | 0.198 | 0.142 | 2.37 | 30.3 | Schoenemann ^3^ and Kappelman ^4^ | 590 | Schoenemann ^3^ and Kappelman ^4^ | 1.8^A^ |
| *H. georgicus* | Dmanisi D2700 | C | 0.156 | 0.112 | 1.05 | 40.0^e^ | Lordkipanidze, *et al*. ^7^ | 600 | Schoenemann ^3^ | 1.7^A^ |
| *A. africanus* | Sts 5 | O | 0.150 | 0.107 | 1.03 | 27.8 | Schoenemann ^3^ | 485 | Schoenemann ^3^ | 2.5^A^ |
| *A. africanus* | Sts 19 | O | 0.152 | 0.109 | 1.09 | 27.2 | Kappelman ^4^ | 436 | Schoenemann ^3^ | 2.5^A^ |
| *A. africanus* | MLD 37 / 38 | O | 0.150 | 0.107 | 1.04 | 27.2 | Kappelman ^4^ | 435 | Schoenemann ^3^ | 3.1^A^ |
| *A. africanus* | STW 53^g^ | C | 0.140 | 0.100 | 0.85 | 27.2 | Kappelman ^4^ | 462 | Holloway, *et al*. ^8^ | ~2.4 – 2.8^B^ |
| *A. africanus* | STW 329^h^ | O | 0.170 | 0.121 | 1.52 | 27.2 | Kappelman ^4^ | 462 | Holloway, *et al*. ^8^ | ~2.4 – 2.8^B^ |
| *A. africanus* | STS 25^h^ | O | 0.157 | 0.112 | 1.20 | 27.2 | Kappelman ^4^ | 462 | Holloway, *et al*. ^8^ | ~2.4 – 2.8^B^ |
| *A. africanus* | STW 98^h^ | O | 0.172 | 0.123 | 1.57 | 27.2 | Kappelman ^4^ | 462 | Holloway, *et al*. ^8^ | ~2.4 – 2.8^B^ |
| *A. africanus* | STW 255^h^ | O | 0.162 | 0.116 | 1.32 | 27.2 | Kappelman ^4^ | 462 | Holloway, *et al*. ^8^ | ~2.4 – 2.8^B^ |
| *A. afarensis* | AL 333-105 | O | 0.165 | 0.118 | 1.38 | 27.2^f^ | Kappelman ^4i^ | 400 | Schoenemann ^3^ | 3.18^E^ |
| *A. afarensis* | AL 166-9 | O | 0.197 | 0.141 | 2.37 | 27.2^f^ | Kappelman ^4i^ | 514 | Herculano-Houzel and Kaas ^9^ | 3.18^E^ |
| *H. naledi* | DH3 (Rising Star) | O | 0.172 | 0.123 | 1.42 | 47.7 | Berger, *et al*. ^10^ | 460 | Berger, *et al*. ^10^ | ~0.285^I^ |

^a^ body mass of LH 18 derived from Omo 1 due to its physiological resemblance as noted by Magori and Day ^11^.

^b^ Ndutu individual was based in Tanzania, and so allocated body mass was estimated by averaging the known body masses of two African *H. heidelbergensis* individuals (Kebwe and Bodo).

^c^ body mass of *H. erectus soloensis* estimated from Sangiran 17 (due to lack of published data), which shares a common endocrinal volume, geographical habitation and age.

^d^ body mass estimated from known body mass of KNM – WT 15000, which shared common age and geographical habitation, although it is noted that KNM ER 42700 (IIIeret) does have significantly smaller endocrinal volume in comparison to mean endocrinal volume for *H. erectus*.

^e^ lower estimated endocrinal volume used owing to subadult developmental stage of Dmanisi D2700 as recommended by [Lordkipanidze *et al.* (2007](#_ENREF_5)).

^f^ body mass of *A. afarensis* derived from body mass estimate of *A. africanus*, as Jungers ^15^ states mininal differences in body size between *Australopithecus* taxa.

^g^ species determined from Kuman and Clarke ^12^ and Clarke ^13^.

^h^ temporal bone specimen species determined from Braga, *et al.* ^14^.

^A^ specimen age determined from Schoenemann ^3^.

^B^ specimen collection from Sterkfontein Member 4 ^16^, and age of specimens identified from McKee, *et al.* ^17^.

^C^ age range according to Lewin and Foley ^5^.

^D “^Bushman – oids” specimens of Mumbwa strata IV aged according to Protsch ^18^.

^E^ specimen age according to Schoenemann ^3^.

^F^ specimen age according to Lahr ^19^.

^G^ mean species age according to Trinkaus ^20^.

^H^ age range according to Sutikna *et al*. ^21^.

^I^ average age according to Dirks *et al*. ^22^.

**References**

1 Aiello, L. C. Five years of *Homo floresiensis*. *Am. J. Phys. Anthropol.* **142**, 167-179 (2010).

2 Falk, D. *et al.* Brain shape in human microcephalics and *Homo floresiensis*. *Proc. Natl. Acad. Sci. U. S. A.* **104**, 2513-2518 (2007).

3 Schoenemann, P. T. in *A Companion to Paleoanthropology*, D. Begun, ed. Pp. 136-164 (Blackwell Publishing Ltd., 2013).

4 Kappelman, J. The evolution of body mass and relative brain size in fossil hominids. *J. Hum. Evol.* **30**, 243-276 (1996).

5 Lewin, R. & Foley, R. A. *Principles of Human Evolution*. (Blackwell Publishing, 2004).

6 Pearson, O. M., Royer, D. F., Grine, F. E. & Fleagle, J. G. A description of the Omo I postcranial skeleton, including newly discovered fossils. *J. Hum. Evol.* **55**, 421-437 (2008).

7 Lordkipanidze, D. *et al.* Postcranial evidence from early *Homo* from Dmanisi, Georgia. *Nature* **449**, 305-310 (2007).

8 Holloway, R. L., Broadfield, D. C. & Yuan, M. S. in *The Human Fossil Record* pp. 295-301 (John Wiley & Sons, Inc., 2005).

9 Herculano-Houzel, S. & Kaas, J. H. Gorilla and orangutan brains conform to the primate cellular scaling rules: implications for human evolution. *Brain, Behav. Evol.* **77**, 33-44 (2011).

10 Berger, L. R. *et al.* *Homo naledi*, a new species of the genus *Homo* from the Dinaledi Chamber, South Africa. *Elife* **4**, e09560 (2015).

11 Magori, C. C. & Day, M. H. Laetoli Hominid 18: an early *Homo sapiens* skull. *J. Hum. Evol.* **12**, 747-753, (1983).

12 Kuman, K. & Clarke, R. J. Stratigraphy, artefact industries and hominid associations for Sterkfontein, Member 5. *J. Hum. Evol.* **38**, 827-847 (2000).

13 Clarke, R. Latest information on Sterkfontein's *Australopithecus* skeleton and a new look at *Australopithecus*. *S. Af. J. Sci.* **104**, 443-449 (2008).

14 Braga, J. *et al.* A new partial temporal bone of a juvenile hominin from the site of Kromdraai B (South Africa). *J. Hum. Evol.* **65**, 447-456 (2013).

15 Jungers, W. L. New estimates of body size in australopithecines. In *Evolutionary History of the “Robust” Australopithecines*, F.E. Grine, ed. Pp. 115-125 (Aldine de Gruyter, 1988).

16 Pickering, T. R., Clarke, R. J. & Moggi-Cecchi, J. Role of carnivores in the accumulation of the Sterkfontein Member 4 hominid assemblage: A taphonomic reassessment of the complete hominid fossil sample (1936–1999). *Am. J. Phys. Anthropol.* **125**, 1-15 (2004).

17 McKee, J. K., Thackeray, J. F. & Berger, L. R. Faunal assemblage seriation of southern African Pliocene and Pleistocene fossil deposits. *Am. J. Phys. Anthropol.* **96**, 235-250 (1995).

18 Protsch, R. Mumbwa: Its absolute chronology and archaeology. *Z. Morphol. Anthropol.* **68**, 1-7 (1977).

19 Lahr, M. M. *Genetics and Fossil Evidence from Modern Human Origins*. (Oxford University Press, 2013).

20 Trinkaus, E. Early modern humans. *Annu. Rev. Anthropol.* **34**, 207-230 (2005).

21 Sutikna, T., Tocheri, M.W., Morwood, M.J., Saptomo, E.W., Jatmiko, Awe, R.D., Wasisto, S., Westaway, K.E., Aubert, M., Li, B., et al. Revised stratigraphy and chronology for *Homo floresiensis* at Liang Bua in Indonesia. *Nature* **532**, 366-369 (2016).

22 Dirks, P., Roberts, E. M., Hilbert-Wolf, H., Kramers, J. D., Hawks, J., Dosseto, A., Duval, M., Elliott, M., Evans, M., Grun, R*. et al.* The age of *Homo naledi* and associated sediments in the Rising Star Cave, South Africa. *Elife* **6,** e24231 (2017).
